# Risk prediction and marker selection in nonsynonymous single nucleotide polymorphisms using whole genome sequencing data

**DOI:** 10.1080/19768354.2020.1860125

**Published:** 2020-12-24

**Authors:** Young-Sup Lee, KyeongHye Won, Donghyun Shin, Jae-Don Oh

**Affiliations:** a Department of Animal Biotechnology, Jeonbuk National University, Jeonju, Republic of Korea; b The Animal Molecular Genetics and Breeding Center, Jeonbuk National University, Jeonju, Republic of Korea; c Department of Agricultural Convergence Technology, Jeonbuk National University, Jeonju, Republic of Korea

**Keywords:** Breeding, deleterious effect, marker selection, nsSNP, risk prediction

## Abstract

Despite the various existing studies about nonsynonymous single nucleotide polymorphisms (nsSNPs), genome-wide studies based on nsSNPs are rare. NsSNPs alter amino acid sequences, affect protein structure and function, and have deleterious effects. By predicting the deleterious effect of nsSNPs, we determined the total risk score per individual. Additionally, the machine learning technique was utilized to find an optimal nsSNP subset that best explains the complete nsSNP effect. A total of 16,100 nsSNPs were selected as the best representatives among 89,519 regressed nsSNPs. In the gene ontology analysis encompassing the 16,100 nsSNPs, DNA metabolic process, chemokine- and immune-related, and reproduction were the most enriched terms. We expect that our risk score prediction and nsSNP marker selection will contribute to future development of extant genome-wide association studies and breeding science more broadly.

## Introduction

Genetic variants can be classified into several categories, including single nucleotide polymorphisms (SNPs), small insertions and deletions, and structural variants (Cooper and Shendure 2011). Among these variants, the majority are SNPs that occur in single bases of DNA sequences. Nonsynonymous SNPs (nsSNP) are an important type of SNP that alter the amino acid sequence as well as potentially affect the protein structure and function (Krawczak et al. 2000; Wu and Jiang 2013).

A number of methods have been proposed for the prediction of deleterious nsSNPs, including the Sorting Intolerant From Tolerant (SIFT) program (Ng and Henikoff 2001), PolyPhen (Galehdari et al. 2013), PolyPhen-2 (Galehdari et al. 2013), variant effect predictor (VEP; (McLaren et al. 2016)), and SnpEff (Cingolani et al. 2012). Deleterious nsSNP prediction is formulated as a binary classification model using diverse genomic data as features to extract deleterious nsSNPs. The classification result can be determined by the aforementioned tools (Wu and Jiang 2013).

We used SIFT scores to estimate the individual risk by nsSNPs and select significant nsSNP markers. SIFT incorporates position-specific information using sequence alignment and is intended for predicting whether an amino acid substitution affects protein function. SIFT converts the alignment into a position-specific scoring matrix and calculates the probability of an amino acid appearing at a specified position. Using this position-specific probability estimation, SIFT assigns a decision rule to make the classification (Ng and Henikoff 2001).

Machine learning (ML) is defined as ‘a field of computer science that evolved from studying pattern recognition and computational learning theory in artificial intelligence and can make predictions on datasets (Taranov; Simon et al. 2016). ML is classified by learning style using the supervised, unsupervised, and semi-supervised learning categories. In supervised data, the input data is referred to as training data. The pre-labeled or categorized data can be applied to the problems of classification or regression algorithms (Taranov).

ML methods consist of computational algorithms to relate all or some of a set of predictors to an outcome. The algorithms attempt to balance two competing interests: bias and variance. In ML contexts, bias is the extent to which the predictions correspond to the true values. Variance represents the sensitivity of the predictions to perturbations in the input data. Even though it is impossible to quantify a model’s bias and variance separately, the two values can be summarized by loss functions. The aim is to reduce both bias and variance simultaneously (Goldstein et al. 2017).

Regression analysis is a statistical tool that models the relationship between quantitative variables using measurements of error from the model (Taranov). There are various studies using regression. The multivariate linear regression model were used for understanding cow evaluations (Mrode and Coffey 2008). The machine learning algorithm using multiple regression were applied to the carcass traits and saleable meat cuts prediction in commercial lambs (Alves et al. 2019).

In this study, we used next-generation sequencing data from a number of pig breeds. One of our goals was to predict individual risk score using the deleterious effect of nsSNPs. Each individual’s risk score was predicted by the nsSNP set and its associated effects. However, the respective nsSNP contributions to the individual risk score were not equivalent. Thus, extraction of the minimal subset of nsSNPs that best explains the entire nsSNP effect, referred to as ‘nsSNP marker selection,’ was critical.

## Materials and methods

### Whole genome sequencing data

The whole genome sequencing data consisted of 106 pigs (Berkshire (BKS) pigs, Duroc (DUR) pigs, Jeju Native pigs (JNP), Jeju Native Black pigs (JNB), Korea Native pigs (KNP), Korea wild boars (KWP), Landrace pigs (LDR), Yorkshire (YKS) pigs, and Yucatan miniature pigs (YMP)). The procedure for producing the sequencing data was as follows: FastQC software was used to perform a quality check on the sequencing data (Brown et al. 2017). The Trimmomatic-0.32 tool was used to remove the potential adapter sequence before aligning the sequence (Bolger et al. 2014). Paired-end sequence reads were mapped to the reference genome (Sscrofa 11.1) from the Ensembl database using the Bowtie2 default setting (Langdon 2015). The following open-source software packages were used for downstream processing and variant calling: Picard tools, SAMtools, and the Genome Analysis Toolkit (GATK) (Li et al. 2009; do Valle et al. 2016). The Picard tools ‘CreateSequenceDictionary’ and ‘MarkDuplicates’ were used to read the reference sequence to write a bam file containing a sequence dictionary and filter potential PCR duplicates, respectively. Index files were created for the reference and bam files using SAMtools. We used the local alignment of sequence reads to correct misalignments using the GATK ‘Realigner-TargetCreator’ and ‘IndelRealigner’ arguments. Base quality score recalibration was utilized to obtain accurate quality scores. The GATK ‘UnifiedGenotyper’ and ‘SelectVariants’ arguments were used with several criteria for calling variants. All variants with 1) a Phread-scaled quality score of < 30, 2) a read depth < 5, 3) an MQ0 (total count across all samples of mapping quality zero reads) > 4, or 4) a Phred-scaled *p*-value > 200 using Fisher’s exact test were filtered to reduce false positive calls due to strand bias. The total number of SNP after SNP calling quality control was 37,410,105. The vcf-merge tool in VCFtools was used to merge all variant calling formats. The number of extracted SNPs was 36,586,008.

### Examination of population structures

To survey the genetic relatedness of the pig samples, we performed principal component analysis (PCA). PCA is a technique for reducing the dimensionality of datasets, increasing interpretability but simultaneously minimizing information loss (Jolliffe et al. 2016). For this purpose, we utilized the Genome-wide Complex Trait Analysis program (Yang et al. 2011). The eigenvector and eigenvalues were computed, and major principal components 1 and 2 (PC1 and PC2) were used to check the separateness of each pig subspecies. Through PC1 and PC2, we examined the genetic relatedness between individuals in the highly-dimensional genomic dataset.

### Extraction and risk score prediction of nsSNPs

To predict whether SNPs were nonsynonymous, we used the SnpEff program with the reference genome version Sscrofa 11.1. SnpEff is a variant annotation and effect prediction tool that can be used to identify differences like amino acid changes (Cingolani et al. 2012). We surveyed the missense variants (nsSNPs) using SnpEff. Additionally, the Ensembl VEP program was used to predict the SIFT scores of the nsSNPs (McLaren et al. 2016). SIFT is a homology-based sequencing tool that does not permit non-resistant amino acid substitutions and predicts whether amino acid substitutions of proteins have phenotypic effects. SIFT is based on the premise that protein evolution correlates with protein function (www.incodom.kr/Interpretation_DB-_sift).

SIFT scores range from 0 to 1. Amino acid substitutions at a given coding sequence with normalized probabilities of < 0.05 are predicted to be damaging, whereas those with normalized probabilities of > 0.05 are predicted to be tolerated. A lower tolerance index indicates a higher functional impact on the translated amino acid residues (Raghav and Sharma 2013).

### Total risk score and linear regression for preprocessing of nsSNP data

Each individual’s total risk score by nsSNPs can be defined by the following equation:

(1) $$Total\_risk\_score_{i}=\;\underset{j}{\sum }G_{ij}(1-SIFT_{j})$$

Where i represents the individual, $G_{ij}$ represents the allele-coded matrix, and (1- SIFT score) is the nsSNP’s predicted deleterious effect. We chose the linear additive model for allele coding, and alternative alleles with amino acid substitutions were coded additively.

For machine learning (ML), nsSNP features should be ordered and selected using some statistical criteria. Here, we set the criteria to be the *p*-values of linear regression. The linear regression model that we used for the preprocessing of nsSNP data and acquisition of regression *p*-values was as follows:

(2) $$Total\_risk\_score_{i}=\;g_{i}\text{β}+\;e_{i}$$

Where $g_{i}$ is the i-individual’s coded alleles, β is the coefficient of the regression model, and $e_{i}$ is the residual error.

### ML for nsSNP feature selection

NsSNPs represent the deleterious effects of translated proteins. These deleterious effects can influence individual survival or risk. Our goal was to select the nsSNPs that significantly affect an individual’s risk among the hundreds of thousands of variants. Thus, we attempted to select nsSNP features through ML (scikit-learn package) (Pedregosa et al. 2011). The root mean squared error (RMSE) statistic was used to identify optimal nsSNPs.

### Quantitative trait loci (QTLs) of the selected nsSNPs

Investigating the traits related to each nsSNP and its deleterious effect can be interesting. To survey the characteristics of the selected nsSNPs related to pig traits, the QTL regions encompassing the selected nsSNPs were inspected. The QTL data were retrieved from the pig QTL database (www.animalgenome.org). The QTL regions in which the ratio of the selected nsSNPs/total nsSNPs was greater than 200-fold were depicted.

## Results

### NsSNP descriptions

**Figure 1.**
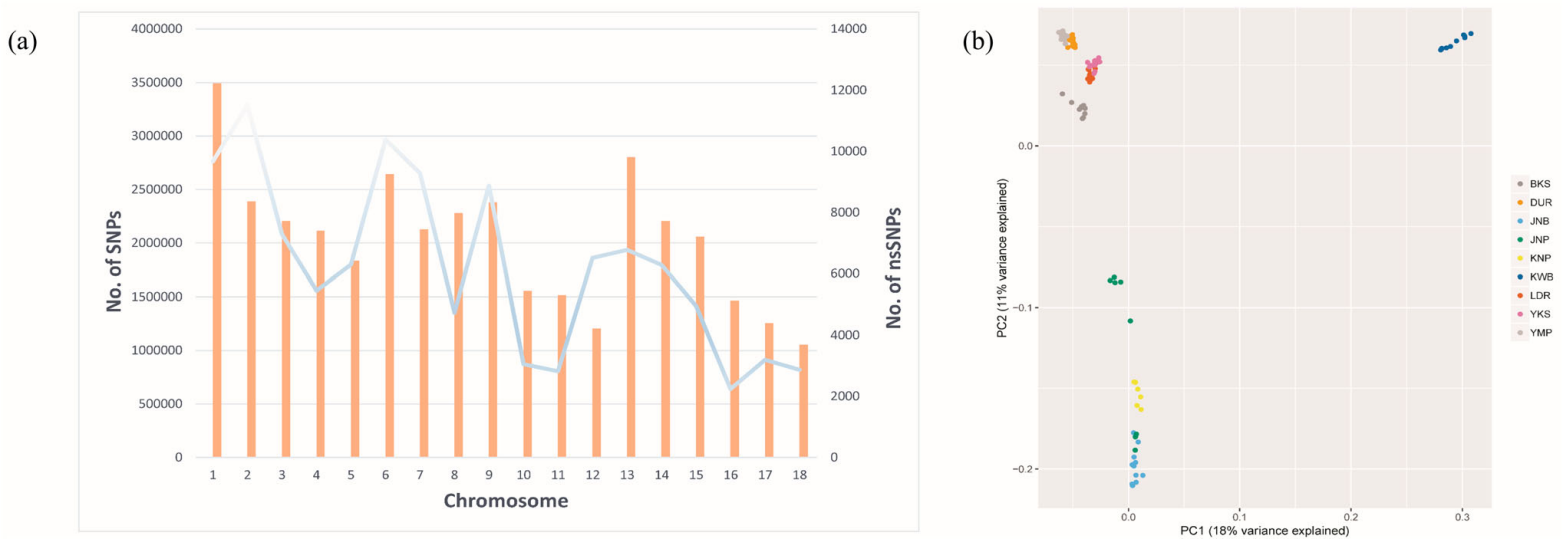
(a) Number of single nucleotide polymorphisms (SNPs) and nonsynonymous SNPs (nsSNPs) across chromosomes. The trends between SNPs and nsSNPs were similar. (b) Principal component analysis (PCA) of pig samples (BKS: Berkshire pigs, DUR: Duroc pigs, JNB: Jeju Native Black pigs, JNP: Jeju Native pigs, KNP: Korea Native pigs, KWB: Korea Wild boar, LDR: Landrace pigs, YKS: Yorkshire pigs, YMP: Yucatan Miniature pigs). Principal component 1 and 2 (PC1 and PC2) explain 18% and 11% of the total variance, respectively.

The number of SNPs per chromosome ranged from 1,055,604–3,492,574. The maximum distance between SNP markers varied from 15,859–107,761, and the average distance (± standard deviation [SD]) spanned 51 (± 124) to 79 (± 111) (Supplementary Table 1). The number of nsSNPs per chromosome ranged from 2,238 to 11,507. The maximum distance varied from 1,281,567–4,015,421, and the average distance (± SD) spanned 9423 (± 53,167) to 35,704 (± 149,954) (Supplementary Table 2). The number of SNP markers and nsSNPs per chromosome tends to co-vary across the board. In this study, the correlation between SNP markers and nsSNPs per chromosome was 0.997 (Figure 1(a)).

### PCA of pig species

PCA was performed to examine the population similarity among 106 pigs. The pig subspecies were BKS, DUR, JNB, JNP, KNP, KWB, LDR, YKS, and YMP. Aside from proximal aggregates between the Landrace and Yorkshire pigs as well as the Duroc and Yucatan miniature pigs, the overall distinctions between breeds were confirmed. PC1 and PC2 explained 18% and 11% of the total variance, respectively (Figure 1(b)).

### VEP: SIFT score prediction

We filtered nsSNPs from the SNP marker data using the SnpEff program. SnpEff predicts variant effects, which are indicated as annotation impacts. These annotation impacts, such as frameshift, stop-gain, and missense variants, are presented in the ANN field of the vcf file (http://snpeff.sourceforge.net). In the VEP program, the nsSNP effects are predicted to be ‘deleterious,’ ‘deleterious with low confidence,’ ‘tolerated,’ or ‘tolerated with low confidence’ based on SIFT score. The number of nsSNPs that were predicted to be ‘deleterious’ was 21,559, and the number that were predicted to be ‘deleterious with low confidence’ was 10,010 (Supplementary Table 3).

### Preprocessing before ML using simple linear regression

The deleterious effect of each nsSNP was represented by a SIFT score. The risk score of each nsSNP was calculated by 1-SIFT score. The total risk score per individual was defined as Eq. 1. The linear regression served as preprocessing for the nsSNP set because the *p*-value in the simple linear regression was utilized as the criteria for sorting the nsSNPs by importance.

**Figure 2.**
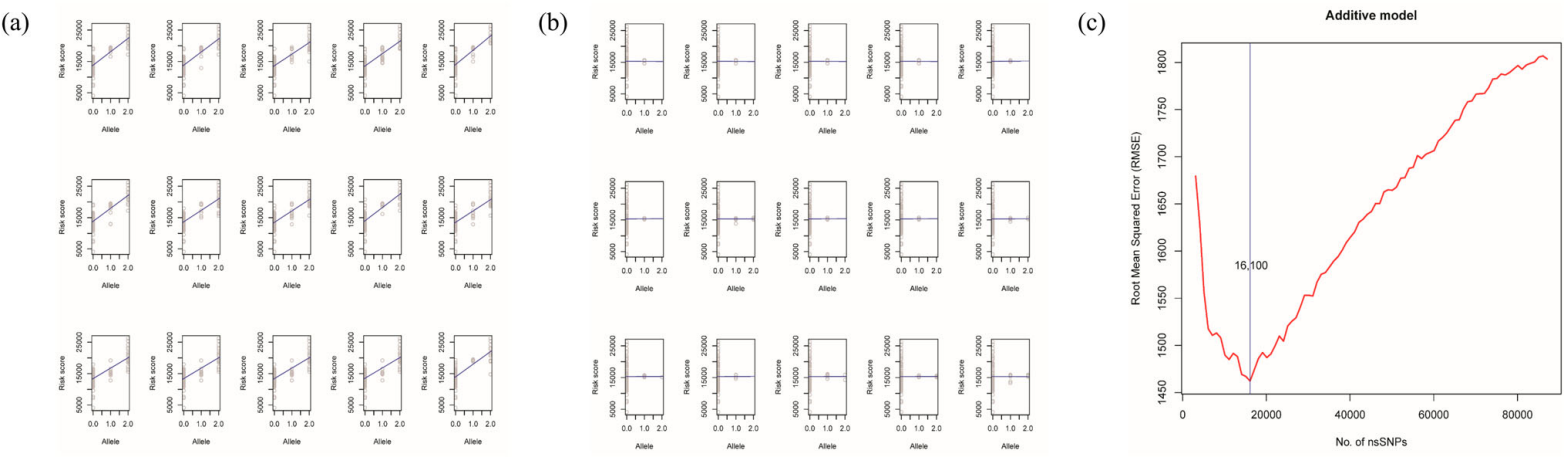
(a, b) Linear regression line of nsSNPs with the lowest *p*-value (a) and highest *p*-value (b). The discrepancy between the two regressions is clear. The response variable was total risk score (see Equation 1), and the deleterious allele was coded additively. (c) Root mean squared error (RMSE) statistic along with the number of ordered nsSNPs. Among all regressed nsSNPs, 16,100 nsSNPs comprised the best subset for explaining the total risk score. These 16,100 nsSNPs can be used as markers for future genomic analyses.

Supplementary Table 4 displays the *p*-value table for preprocessing using simple linear regression. The number of nsSNPs with a *p*-value < 10^−12^, between 10^−10^ and 10^−12^, and between 10^−4^ and 10^−6^ was 1,583, 1430, and 7,456, respectively, among the 89,519 total regressed nsSNPs. Figure 2(a and b) illustrate the nsSNP regression plot with the lowest and highest *p*-values. The discrepancy between the two cases was evident.

### ML and multiple linear regression

**Figure 3.**
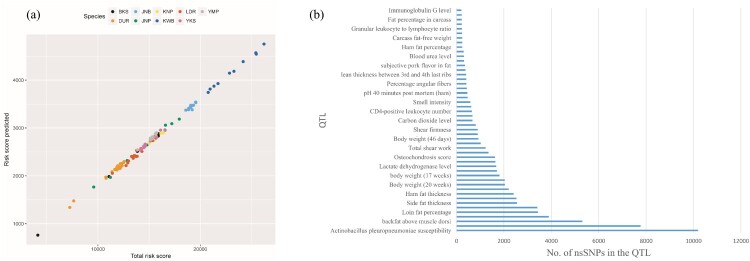
(a) Plot of predicted risk scores (from the 16,100 nsSNP markers) against total risk scores. (b) Quantitative trait loci (QTLs) across the number of nsSNPs. The QTL regions in which the ratio of the selected nsSNPs (16,100 nsSNPs)/total nsSNPs was greater than 200-fold were chosen and depicted. Many of the nsSNPs belonged to the body weight and fat QTLs.

We performed multiple linear regressions after preprocessing. The scikit-learn package was utilized for ML (Pedregosa et al. 2011), and the RMSE statistic was used for feature selection. The plot of the RMSE against the number of nsSNPs resembled the ML theoretic curve (Anderson et al. 2018). The RMSE across the number of nsSNPs showed a minimum value at 16,100 nsSNPs (Figure 2(c)). We determined that 16,100 nsSNPs comprised the optimal subset of markers that best explains the total risk. The risk score predicted against the total risk score displayed linearity (Figure 3(a)). The nine breeds are clustered into each breed as seen in PCA analysis. The total risk score was clustered with respect to each breed. Figure 3(b) demonstrates the number of nsSNPs in each QTL. The 16,100 significant nsSNPs were abundant in the fat-related and body weight QTLs. The supplementary provides the fat and body weight QTLs encompassing nsSNPs.

### Gene ontology (GO) analysis of selected nsSNP markers

GO analysis was performed using the selected 16,100 nsSNP markers (Table 1). The most enriched GO terms were DNA metabolic process (GO:0006259), chemokine- and immune-related (GO:0002682 and GO:0032602), and reproduction (GO:0000003). Among these GOs, the notable genes were as follows: N-myc and STAT interactor (NMI; GO:0002682∼regulation of immune system process; lowest *p*-value: 2^−16^), toll-like receptor (TLR3; GO:0002682∼regulation of immune system process; 4^−15^), calcium- and integrin-binding 1 (CIB1; GO:0030307∼positive regulation of cell growth; 1^−18^), erythropoietin-producing hepatoma receptor A2 (EPHA2; GO:0032602∼chemokine production; 1^−14^), and colony stimulating factor 2 (CSF2; GO:0006259∼DNA metabolic process; 1^−15^).

**Table 1.** Gene ontology (GO) analysis encompassing the 16,100 selected nonsynonymous single nucleotide polymorphism (nsSNP) markers. The most notable GO terms were DNA metabolic process (GO:0006259), chemokine and immune-related (GO:0002682 and GO:0032602), and reproduction (GO:0000003).

Term	Count	*P*-Value	Genes
GO:0006302∼double-strand break repair	10	0.005	*DCLRE1C, SLX4, XRCC2, FIGNL1, DTX3L, EME2, SLF1, PRKDC, BRCA1, SETX*
GO:0007010∼cytoskeleton organization	30	0.006	*ABLIM1, GFAP, XRCC2, CCDC88A, CEP120, PLEK, FIGNL1, NF1, ERMN, HAUS1, KIF18A, LRGUK, CXADR, CEP152, PCLO, SIGLEC15, BRCA1, WEE1, DSTN, TMEM67, CORO1A, LARP4, SLK, DNAAF2, SVIL, STRIP2, WIPF3, ANTXR1, EMP2, CIB1*
GO:0000002∼mitochondrial genome maintenance	4	0.008	*OPA1, PIF1, MRPL39, DNAJA3*
GO:0006259∼DNA metabolic process	25	0.008	*ESCO1, CSF2, XRCC2, REV1, CCDC88A, FIGNL1, EME2, DTX3L, PIF1, SLF1, PRKDC, MEIOB, CCT2, MBD1, BRCA1, SETX, DCLRE1C, SLX4, FANCI, TDP1, POLD2, PDGFC, PMS1, DNAJA3, ASTE1*
GO:0002682∼regulation of immune system process	29	0.010	*NMI, C6, MRPS10, TLR3, CACNB4, APOD, DNAAF2, LEO1, ANO6, DNAJA3, CYP19A1, CIB1, HAVCR2, SELP, IKZF3, SOX13, KLF13, IL1RL1, CD3E, NF1, ZNF189, C4BPA, NLRP3, REEP2, SIGLEC15, ECM1, GPR33, CORO1A, PLA2G7*
GO:0006281∼DNA repair	16	0.011	*XRCC2, REV1, FIGNL1, PIF1, EME2, DTX3L, SLF1, PRKDC, BRCA1, SETX, DCLRE1C, SLX4, FANCI, TDP1, ASTE1, PMS1*
GO:0032606∼type I interferon production	5	0.012	*HAVCR2, CSF2, NMI, ZC3HAV1, TLR3*
GO:0022607∼cellular component assembly	51	0.013	*XRCC2, CCT2, SETX, BDNF, SLK, APOD, NUBP2, PDGFC, ANO6, VWA2, SH3PXD2B, CEP295, CCDC88A, OPA1, MPP7, PADI4, NLRP3, CEP152, PCLO, TIMM21, TRPM1, WEE2, TMEM67, ADSL, USH1C, EMP2, EIF2AK3, IFT74, ABLIM1, CSF2, CEP120, MRPS11, HAUS1, SLF1, NPRL3, HJURP, DNAAF2, WIPF3, GEMIN7, GEMIN5, IFT140, SELP, RPSA, PLEK, SPTBN5, LRGUK, EPHA2, CORO1A, PKP4, ANTXR1, ATG16L2*
GO:0044085∼cellular component biogenesis	56	0.014	*XRCC2, CCT2, SETX, BDNF, SLK, APOD, NUBP2, PDGFC, ANO6, VWA2, SH3PXD2B, CEP295, CCDC88A, OPA1, NECTIN1, MPP7, PADI4, NLRP3, CEP152, PCLO, TIMM21, TRPM1, WEE2, TMEM67, ADSL, USH1C, EIF2AK3, EMP2, IFT74, ABLIM1, CSF2, CEP120, MRPS11, HAUS1, SLF1, NPRL3, DNAAF2, HJURP, WIPF3, WDR12, GEMIN7, GEMIN5, IFT140, SELP, RPSA, PLEK, NOC4L, SPTBN5, LRGUK, HEATR1, EPHA2, CORO1A, PKP4, NOP58, ANTXR1, ATG16L2*
GO:0032602∼chemokine production	5	0.014	*HAVCR2, APOD, IL1RL1, TLR3, EPHA2*
GO:0007229∼integrin-mediated signaling pathway	6	0.016	*ADAM10, PLEK, FUT8, ADAMTS20, ITGAE, EMP2*
GO:0032642∼regulation of chemokine production	5	0.016	*HAVCR2, APOD, IL1RL1, TLR3, EPHA2*
GO:0045765∼regulation of angiogenesis	9	0.017	*SASH1, C6, NF1, SULF1, EMP2, ECM1, EPHA2, BRCA1, CIB1*
GO:0022414∼reproductive process	28	0.018	*CSF2, XRCC2, PRKDC, CCT2, PRDX3, CXADR, DPY19L2, SLX4, PKD1, EIF2B2, CYP19A1, CIB1, HAVCR2, EME2, KIF18A, MEIOB, NPR2, LRGUK, WEE2, PSP-II, SPAI-2, UMODL1, SGO2, SERPINB5, DLD, SULF1, ANTXR1, EMP2*
GO:0000003∼reproduction	28	0.019	*CSF2, XRCC2, PRKDC, CCT2, PRDX3, CXADR, DPY19L2, SLX4, PKD1, EIF2B2, CYP19A1, CIB1, HAVCR2, EME2, KIF18A, MEIOB, NPR2, LRGUK, WEE2, PSP-II, SPAI-2, UMODL1, SGO2, SERPINB5, DLD, SULF1, ANTXR1, EMP2*
GO:0050900∼leukocyte migration	11	0.023	*GPR33, SELP, HRH1, CORO1A, APOD, PLA2G7, JAML, CXADR, ANO6, ECM1, CYP19A1*
GO:0044702∼single organism reproductive process	26	0.024	*HAVCR2, CSF2, XRCC2, EME2, KIF18A, PRKDC, MEIOB, LRGUK, NPR2, CCT2, PRDX3, CXADR, DPY19L2, WEE2, PSP-II, SLX4, UMODL1, SGO2, SERPINB5, SULF1, DLD, PKD1, EIF2B2, EMP2, CIB1, CYP19A1*
GO:0050790∼regulation of catalytic activity	35	0.025	*PPP1R14D, APH1B, CNPPD1, CCT2, PPP6R3, PRDX3, IQGAP1, ARHGAP21, ITIH1, SERPINA6, PKD1, SCG5, ITIH2, PDGFC, TBC1D9B, CIB1, SASH1, CCDC88A, PLEK, PIF1, NF1, NLRP3, ECM1, EPHA2, RUBCN, WEE2, SPAI-2, PSME1, PKP4, CYFIP2, LRRC66, SEMA4D, ANTXR1, EMP2, NEK5*
GO:0030307∼positive regulation of cell growth	6	0.025	*BDNF, ADNP2, MACF1, NEDD4L, SEMA4D, CIB1*
GO:0007166∼cell surface receptor signaling pathway	48	0.026	*ADGRF3, CSF2, NMI, ADGRF1, FUT8, ADGRF4, ITGAE, IL19, APH1B, MKNK2, TLR3, CACNB4, IQGAP1, SETX, FAM83B, BDNF, TSPAN33, MACF1, APOD, WDR12, ZNF106, PDGFC, DEPDC1B, FRS2, ANO6, CIB1, SASH1, ADAM10, PLEK, ADAMTS20, CD3E, NF1, CILP, ADGRG5, NPR2, ECM1, BRCA1, TRPM1, GPR33, DKK3, CORO1A, KCP, SULF1, LRRC66, SEMA4D, EMP2, AKAP3, IFT74*
GO:0001816∼cytokine production	16	0.027	*HAVCR2, CSF2, LIPA, NMI, ZC3HAV1, CD3E, IL1RL1, IL19, TLR3, NLRP3, ARFGEF2, EPHA2, BRCA1, APOD, SULF1, EIF2AK3*
GO:0031122∼cytoplasmic microtubule organization	4	0.027	*CEP120, SLK, FIGNL1, CIB1*
GO:1901342∼regulation of vasculature development	9	0.028	*SASH1, C6, NF1, SULF1, EMP2, ECM1, EPHA2, BRCA1, CIB1*

NMI encodes a protein that interacts with N-MYC and C-MYC. These proteins are two members of the oncogene MYC family. NMI also interacts with all STATs, except STAT2, and augments STAT-mediated transcription in response to the cytokines interleukin-2 (IL-2) and interferon-gamma (IFN-γ). NMI is linked to the JAK-STAT cascade and negative regulation of type I IFN production GO terms. TLR3 encodes a member of the toll-like receptor family, which plays a key role in pathogen recognition and activation of innate immunity. They recognize pathogen-associated molecular patterns and mediate the production of cytokines that are necessary for immunity development. CIB1 encodes a member of the EF-hand domain-containing calcium-binding superfamily, which interacts with many other proteins. These include platelet integrin alpha-IIb-beta-3, DNA-dependent protein kinase, presenilin-2, focal adhesion kinase, protein kinase D, and p21-activated kinase. CIB1 is linked to the type II diabetes mellitus and leukocyte count GO terms. EPHA2 belongs to the ephrin receptor superfamily of the protein tyrosine kinase family, which has been implicated in mediating developmental events, particularly in the nervous system. EPHA2 is linked to the protein kinase activity and protein tyrosine kinase activity GO terms. CSF2 encodes a cytokine that controls the production, differentiation, and function of granulocytes and macrophages and is associated with eosinophil count and inflammatory bowel disease. CSF2 is linked to the cytokine activity and growth factor activity GO terms (www.genecards.org).

## Discussion

### Characteristics of the method

Our method was based on the ‘from genome to genome’ concept rather than ‘from phenotype to genome’ like classic genome-wide association studies (GWASs) (Catchpole et al. 2008). All of the information in our approach originates from the genome because each nsSNP as well as its effect (SIFT score), total risk score, regression coefficient, linear regression *p*-value, and ML stemmed from a self-genome basis. In particular, the total risk score of an individual (the regression response variable) was based on the SIFT score of each nsSNP. Thus, the accuracy of our analysis was primarily dependent on SIFT score estimation.

### Individual risk score and marker selection

The total risk score per individual reflects the risks due to nsSNP deleterious effects. It can serve as the parameter of each individual’s genetic risk as a result of translated protein structures and functions. In breeding science, prediction of the genomic estimated breeding value is the main goal (Pryce et al. 2011). However, with consideration of the infinitesimal deleterious SNP effects and nsSNP accumulation in breeding lineages, organismal high risk can be generally be avoided in the long-term. Thus, nsSNP marker selection using risk score prediction should be utilized on its own. Furthermore, nsSNP markers can be used in future GWASs as an important marker set.

### ML and multiple linear regression

In the scikit-learn package, multiple linear regression can be accompanied by nsSNP marker selection. Before performing multiple linear regression, we attempted to preprocess nsSNP sorting by *p*-value from the simple linear regression. This process ensured that the nsSNPs were arranged according to the lowest *p*-value order rather than deleterious effect. Thus, the nsSNP disposition was identified using a significant order for the regression rather than by adverse effects. After ML, the number of markers that best reflected the data in the nsSNP set was 16,100, which included not only the deleterious effect markers but also a large number of tolerated effect nsSNPs (only 4,466 deleterious nsSNPs out of 16,100).

## Conclusion

NsSNPs have deleterious effects and are represented by SIFT scores. Given this knowledge, we predicted the total risk score using the SIFT scores of nsSNPs in various pig breeds. Furthermore, nsSNP markers that best explained the total risk score were selected using ML. In addition to the utility of the total risk score, the selected nsSNPs can serve as SNP markers for future GWASs and breeding research.

## Data Availability

The datasets analyzed during the current study are not publicly available due intellectual property considerations but are available from the corresponding author on reasonable request.
